# The closure of Wyoming’s Program of All-Inclusive Care for the Elderly (PACE): Qualitative analysis of the impact on social isolation and loneliness

**DOI:** 10.3389/fpubh.2024.1426100

**Published:** 2024-11-14

**Authors:** Barbara S. Dabrowski, Christine L. McKibbin, Gregory W. O'Barr, Elizabeth L. Punke, Abby L. Teply, Kathryn A. Richardson, Catherine P. Carrico

**Affiliations:** ^1^Department of Psychology, College of Arts and Sciences, University of Wyoming, Laramie, WY, United States; ^2^Cheyenne Regional Medical Center, Behavioral Health and Population Health Services, Cheyenne, WY, United States

**Keywords:** aging, loneliness, social isolation, older adults, chronic conditions, social determinants of health, Program of All-Inclusive Care for the Elderly (PACE)

## Abstract

**Introduction:**

Social isolation and loneliness are global public health concerns experienced among older adults which are commonly associated with negative physical, psychological, and social outcomes. The healthcare system has an opportunity to identify and address social isolation and loneliness in older adults. The Program of All-Inclusive Care for the Elderly (PACE) was developed to serve comprehensive social needs along with medical and behavioral needs of older adults who qualify for long-term care while still living in the community. In 2021, due to state budget reductions, Wyoming’s only PACE program (WY PACE) closed, resulting in the discharge of all participants and loss of social engagement opportunities provided by this program. The objectives of this evaluation were to (1) examine the impact of the WY PACE closure on isolation and loneliness, (2) identify how older adults adapted to the loss of services addressing isolation and loneliness, and (3) identify needs for future interventions to address isolation among clients who experienced loss of supportive programs.

**Methods:**

A mixed-methods design was used to facilitate understanding of qualitative findings while also conducting quantitative analyses to provide context for qualitative responses. Participants included 17 individuals who were either former PACE participants or their caregivers. Participants (*n* = 12; *M* = 74 years old) were predominantly non-Hispanic White (*n* = 8, 66%) and cisgender female (*n* = 7, 58%). Caregivers of participants (*n* = 5; *M* = 63 years old) were predominantly Hispanic, Latino, or of Spanish origin (*n* = 3, 60%) and cisgender female (*n* = 4, 80%).

**Results:**

A mixed-methods design was used to facilitate understanding of qualitative findings while also conducting quantitative analyses to provide context for qualitative responses. Participants included 17 individuals who were either former PACE participants or their caregivers. Participants (*n* = 12; *M* = 74 years old) were predominantly non-Hispanic White (*n* = 8, 66%) and cisgender female (*n* = 7, 58%). Caregivers of participants (*n* = 5; *M* = 63 years old) were predominantly Hispanic, Latino, or of Spanish origin (*n* = 3, 60%) and cisgender female (*n* = 4, 80%).

**Discussion:**

This evaluation provided preliminary insight into the impacts of the loss of programs like WY PACE on social isolation and loneliness. Creative solutions to maintain social engagement of this vulnerable population are needed.

## 1 Introduction

Social isolation (i.e., the objective state of having few social relationships or limited social contact with others) and loneliness (i.e., the subjective feeling of being isolated from others) are global public health issues that have adverse psychological, physical, and social effects (1, 2). Isolation and loneliness are particularly detrimental to older adults, contributing to increased falls, cardiovascular disease, serious illness, functional decline, and malnutrition (3). Social isolation and loneliness have also been associated with heightened stress (4), increased depressive symptoms (5), and increased suicidal ideation (6) as well as death by suicide (7) among older adults.

The issues of social isolation and loneliness are increasingly prevalent, worldwide (8). In the United States, Cudjoe and colleagues found that approximately 24% of community-dwelling older adults 65 years and older are socially isolated (9). Further, data collected as part of the U.S. Health and Retirement Study demonstrated that 43% of individuals aged 60 and older reported feelings of loneliness (10). Similar rates of social isolation and loneliness were also found internationally. In the United Kingdom, the Age UK (11) organization estimated that nearly 1.4 million older adults are severely lonely. Similarly, data gathered from the Chinese Longitudinal Healthy Longevity Survey found that nearly 28% of older adults in China reported feeling lonely (12). Taken together, these findings highlight the global impact of social isolation and loneliness on older adults.

Comprehensive healthcare models are strategically positioned to combat social isolation and loneliness among older adults. The opportunity for healthcare systems to identify and address social isolation and loneliness was recognized as early as 1985 when Jones et al. (13) indicated that older adults, due to increasing physical health needs, tend to visit healthcare providers more frequently than other sectors. The researchers emphasized that, due to this frequent contact, medical providers are in a key position to identify and address social isolation and loneliness. One example of a comprehensive program that, by its structure, may address isolation and loneliness is the Program of All-Inclusive Care for the Elderly (PACE). The first PACE program, originally established as On Lok Senior Health Services in 1971 in San Francisco’s Chinatown, aimed to provide an alternative to nursing home care by offering a range of services, including adult day care, medical care, and social support (14). The model’s success led to federal Medicaid and Medicare waivers in 1986, enabling the nationwide replication of this comprehensive care model for older adults (14).

PACE was specifically designed to meet the increasing needs of low-income older adults with chronic conditions who struggle with daily activities such as bathing, dressing, toileting, and eating (15). These challenges pose barriers to independent living and contribute to a high reliance on nursing homes (16). PACE was designed to reduce the need for nursing home care by providing community-based support that enables older adults to remain in their homes for as long as possible (14, 17). A goal of PACE is to enhance the quality of life and independence of older adults through comprehensive, wrap-around services that address both medical and social needs (15). The primary mechanism driving the PACE model is the interdisciplinary team, which includes an interprofessional team of healthcare providers, behavioral health workers, and other helping professionals working collaboratively to create individualized care plans (14). These teams monitor health status, coordinate medical treatments, and provide in-home assistance, adapting care as participants’ needs change (15).

To fund these services, PACE receives monthly capitation payments from Medicare and Medicaid, which are fixed amounts paid per patient to cover their healthcare costs each month (18). If a participant is not eligible for Medicaid, then participants are responsible for paying privately for the portion that Medicaid would typically cover (18). Most of the individuals who participate in PACE programs are dually eligible for both Medicare and Medicaid (14). These financial resources enable the interdisciplinary team to offer not only medical care but also emotional and social support for participating older adults. With a defined service area, clients have access to transportation, meals, health services, and a community of others in the program with whom to socially interact. Maintaining social connectedness is an important part of aging (19), and PACE provides the opportunity for clients to address social isolation and loneliness indirectly and directly, as they meet with others, share a meal, and socially connect with their local community. Additionally, participants engage in a variety of structured social activities, including games, community outings, volunteer work, and arts and crafts. These activities are scheduled between their medical appointments at PACE, further integrating social interaction into their routine care. The short-term outcomes of PACE include increased access to comprehensive healthcare, improved management of chronic conditions, and opportunities for increased socialization (15). Over time, PACE programs have been found to contribute to long-term outcomes such as reduced hospitalizations and fewer nursing home admissions (20).

In October of 2021, Wyoming state budget cuts eliminated critical Medicaid funding for Wyoming’s only PACE program. The elimination of Wyoming (WY) PACE, deemed necessary because of “historic declines in state revenues,” according to the Wyoming Department of Health (21), was intended to save approximately $2 million in state funding. Therefore, all 139 members were discharged from the WY PACE service, and the program closed its doors in October of 2021. Important opportunities for former WY PACE members to socially engage with their care team and fellow members were eliminated. While one evaluation of a PACE closure in another state was designed to document negative impacts on health outcomes, emergency department use, and hospitalization (22), little is known about the effects of the program closure on social isolation and loneliness or what program features may be necessary to socially sustain individuals like those served by PACE.

The purpose of the current evaluation was threefold: (1) to describe the outcomes of the WY PACE closure on social isolation and loneliness among former WY PACE participants; (2) to describe ways that former WY PACE participants adapted to the loss of social engagement services (e.g., adult day services and socialization opportunities provided by WY PACE); and (3) to identify the needs and preferences of former WY PACE participants for future interventions to address social isolation and loneliness. Importantly, response and adaptation to program closure, as well as future needs, may vary by physical vulnerability. Therefore, qualitative interviews were contextualized based on risk for nursing home placement. A qualitative approach within a mixed-methods design is justified when there is limited published data in a particular area of inquiry. In this case, there are limited, published outcomes evaluation data representing enrolled participants’ experiences following a PACE program closure and even less data regarding social impacts of program closure. Therefore, an examination of the effects of WY PACE closure on the social worlds of WY PACE participants was warranted.

## 2 Methods

### 2.1 Design

A mixed-method design was used for this retrospective outcomes evaluation, in which both qualitative data and quantitative data were included in the data collection and analysis process (23, 24). Qualitative methods were used to ask open-ended and exploratory questions of the participants to better understand the phenomena of interest (25). Quantitative self-report data, reflecting physical and psychosocial health, were also collected to characterize the sample and add context to the qualitative data gathered through interviews. A qualitative approach within a mixed-methods design is particularly beneficial when there is limited knowledge in an area. In this case, there are limited evaluation findings related to health outcomes among formerly enrolled participants following a PACE program closure (22), and, little if any published evaluation findings describing the impact of PACE closure on social isolation and loneliness among previously registered participants. Therefore, the use of qualitative data in the outcomes evaluation allowed for the development of a rich understanding of the experiences of WY PACE participants in the year following the WY PACE closure.

### 2.2 Participants

To be included in the evaluation, participants were required to be previously enrolled in the WY PACE program at Cheyenne Regional Medical Center (CRMC) at the time of program closure, be discharged from the program at the time of WY PACE closure, be age 55 or older at the time of WY PACE closure, be residing in the prior WY PACE catchment area, and be participants who sought outpatient primary care with their same WY PACE provider. Participants were also included if they were caregivers of former WY PACE participants. Participants were excluded if they were living in a skilled nursing facility either while enrolled in the WY PACE program or after closure of the program, as their social needs were likely met by the facility. Therefore, 47 of the original 139 WY PACE participants were eligible for this evaluation. Out of the 47 participants contacted, 12 former WY PACE participants and five caregivers participated in this evaluation.

### 2.3 Procedure

All participants were recruited by a staff member of CRMC or their designated staff/interns through an initial letter, followed by telephone outreach, notifying them about the evaluation at one-year post WY PACE closure. First, a member of CRMC reviewed participant medical records to determine eligibility. A former member of the PACE team reached out via a mailed letter to notify potential participants and their caregivers about the evaluation and that a member of the evaluation team would contact them by telephone. Two weeks following the informational letter, a member of the team contacted eligible individuals or their caregivers, reintroduced the nature of the evaluation, and invited them to participate. Participants who agreed to the evaluation were scheduled for a 60-80-minute meeting with a trained interviewer. To ensure a participant’s ability to consent, the informed consent document was read to the participant and questions posed regarding the nature of the evaluation, risks and benefits of participation, and right to withdraw. For any participant who failed to answer these questions correctly, the relevant portion of the consent form was reread, and the questions were posed up to three times. If a caregiver indicated that a participant would be unable to complete the interview, the interview was then only conducted with the caregiver. All but one participant provided informed consent. In that case, the interview was only conducted with a consenting caregiver. During the interviews, participants and caregivers who were present were asked if they were willing to be audio-taped. Following informed consent, participants were instructed to complete self-report measures reflecting their current situation and experience, which served to contextualize qualitative data. These measures included a sociodemographic questionnaire, and measures of loneliness, isolation, depressive symptoms, and physical health. Participants who were unable to complete the measures (e.g., participants who were unable to write) were given the measures orally by the interviewer or their caregiver. Following completion of all measures, participants and caregivers who were present were asked to participate in a semi-structured interview. The interviewer asked questions about the experiences of the former WY PACE participants when the program closed, how it impacted their social worlds, and how they adapted to the loss of program benefits. The interviewer also provided participants the opportunity to discuss their recommendations for an intervention to address social isolation and loneliness for older adults with chronic illnesses. After completing the measures and the interview, participants were thanked and compensated twenty dollars for their time. Following the interview, the evaluation team reviewed Plans of Care within participants’ medical records and extracted relevant medical and psychosocial data that was recorded at the time of WY PACE closure.

#### 2.3.1 Qualitative interview

A set of nine qualitative interview questions were developed by the evaluation team to understand the experiences of the participants who were discharged from WY PACE as a result of program closure over the prior year. Questions were designed to explore the social experiences of former WY PACE participants in response to program loss as well as their recommendations for addressing social isolation and loneliness among people who are former WY PACE participants (see Table 1). The interviewer asked the series of general questions and specific prompts were added or changed to explore particular aspects of social isolation and loneliness. All materials utilized in this evaluation, including the qualitative interview and quantitative data, can be made available upon request.

**Table 1:** Qualitative interview questions.

Tell me about your experiences with the PACE program. Can you tell me about the time when you first heard about the closure of PACE? What does the closure of the PACE program mean for you now? Tell me about how you socialized at PACE. How has the closure of PACE impacted your mental health and/or well-being? What was the most meaningful or valuable part of the PACE program for you? Tell me about what you did to deal with the changes in your social life after PACE closed. What are some current recommendations or ideas you have for the state of Wyoming to implement regarding care for older adults? If we were to develop something that would make a difference in your social life, what do you think it should be? What do you think we should do?

### 2.4 Measures

#### 2.4.1 Sociodemographic and clinical measures for contextualizing qualitative findings

##### 2.4.1.1 Demographic questionnaire

Demographic information was collected about WY PACE participants and caregivers who were present. This demographic information included information about their age, biological sex, gender identity, ethnicity, race, marital status, housing situation, income, current household membership, and presence or absence of a caregiver in the home.

##### 2.4.1.2 Plan of care at program closure

Plans of Care at the time of WY PACE closure comprised information regarding participants’ living situation, cognitive functioning, depressive symptoms (i.e., Geriatric Depression Scale Score; GDS; 26), presence of chronic and acute illnesses, and psychosocial problems such as social isolation. In addition, at the point of closure, the WY PACE interprofessional team developed a risk score to categorize patients by their risk for nursing home placement and included that risk score in the final Plans of Care. Despite all participants qualifying for nursing home care, their level of risk for nursing home placement varied. Risk was categorized in the following way: (1) participants are currently placed in a nursing home facility or assisted living facility for custodial care, where they are no longer able to live safely in the community; (2) participants are in immediate jeopardy of placement in nursing care within the next six months, meaning they would likely not be able to live safely in the community without WY PACE services; (3) participants are medically stable with ongoing home caregiver support but will need strong medical management; and (4) participants are in no immediate jeopardy when receiving home and community-based services.

##### 2.4.1.3 Physical health, mental health, and psychosocial measures

To contextualize the interview data, several standardized measures were employed to assess social, mental, and physical functioning at the time of the interview. Social support was measured by both the Berkman-Syme Social Network Index (26) and the Multidimensional Scale of Perceived Social Support (MSPSS; 27). Loneliness was assessed using the Revised UCLA Loneliness Scale (R-UCLA; 28). Depression was evaluated using the GDS (29). Physical health status was characterized by the Short Form-36 Health Survey (SF-36; 30).

### 2.5 Data analysis

#### 2.5.1 Qualitative coding

Two raters (BSD and CLM) conducted a content analysis using emergent thematic coding to determine the underlying themes (31). An inductive thematic analysis approach was chosen, which allows patterns to emerge directly from the data without using a pre-existing theoretical framework (32). The purpose of this evaluation was to explore participants’ experiences using a bottom-up approach rather than to evaluate outcomes based on a predefined model (33). The raters followed a three-step procedure (i.e., open coding, axial coding, and selective coding) according to Corbin and Strauss (34). First, the raters read each transcript independently and created a list of open codes for each passage in the transcripts. Second, axial coding was conducted, where the open codes were combined into fewer, more comprehensive categories. Third, selective coding was completed, where the raters identified core categories or central themes that best represent the essence of the data. The reliability of coding was deemed acceptable once discrepancies were discussed and mutually agreed upon and a 100% agreement between raters was reached across all themes (31). Upon finding emerging themes, the raters independently assessed the frequency of occurrence of the categories. In accordance with the recommendations of Francis and colleagues (35), an agreement rate of 75% or higher was achieved among raters for all codes. To assess data saturation, a data saturation table containing all codes from the codebook was created. Data saturation was achieved with 17 interviews. No additional codes emerged after the 15th participant was interviewed.

## 3 Results

### 3.1 Sample characteristics

A total of 17 participants (i.e., 12 former WY PACE participants and 5 caregivers) provided informed consent and completed the qualitative interview and quantitative measures. Former WY PACE participants were an average age of 74 years old and were primarily non-Hispanic White cisgender females (see Table 2). Caregivers, with an average age of 63 years old, were predominantly Hispanic, Latino, or of Spanish origin, cisgender females, and living with a spouse or partner (see Table 3). Of the 12 former WY PACE participants, four were unable to independently complete the quantitative measures. Participants who were unable to complete measures independently had the measures presented to them orally by a researcher.

**Table 2:** Characteristics of former PACE participants.

Characteristics	*M ± SD* (range)	*n* (%)
Age	74 ± 9.5 (61–88)	
Risk score		
Immediate jeopardy, may need skilled nursing facility within six months		3 (25%)
Medically stable with ongoing home caregiver support		3 (25%)
No immediate jeopardy with waiver support		6 (50%)
Sex (female)		7 (58%)
Gender (cisgender)		12 (100%)
Primary language (English)		12 (100%)
Ethnicity		
Non-Hispanic		8 (66.7%)
Hispanic (Mexican, Mexican American, Chicano)		3 (25%)
Hispanic (another Hispanic, Latino, or Spanish Origin)		1 (8.3%)
Race (White)		12 (100%)
Education		
Some elementary, middle, or high school		2 (16.7%)
High school graduate or GED		4 (33.3%)
Some college or technical school		4 (33.3%)
College 4 years or more		2 (16.7%)
Income		
Below 10,000		4 (33.3%)
10,000–30,000		7 (58.3%)
30,000–70,000		1 (8.3%)
Marital status		
Married		4 (33.3%)
Divorced		5 (41.7%)
Widowed		1 (8.3%)
Never married		2 (16.7%)
Household membership		
Lives alone		6 (50%)
Lives with spouse or partner		4 (33.3%)
Lives with family members/caregiver		2 (16.7%)
Living situation		
Rent apartment/condo		5 (41.7%)
Owns house/townhouse		6 (50%)
Houseless		1 (8.3%)

*N* = 12, Mean (M), Standard deviation (SD), sample size (*n*), Percentage (%).

**Table 3:** Characteristics of caregivers of former PACE participants.

Characteristics	*M ± SD* (range)	*n* (%)
Age	74 ± 9.5 (61–88)	
Sex (female)		4 (80%)
Gender (Cisgender)		5 (100%)
Primary language (English)		5 (100%)
Ethnicity		
Non-Hispanic		2 (40%)
Hispanic (Mexican, Mexican American, Chicano)		1 (20%)
Hispanic (another Hispanic, Latino, or Spanish Origin)		2 (40%)
Race (White)		5 (100%)
Education		
Some elementary, middle, or high school		1 (20%)
High school graduate or GED		1 (20%)
Some college or technical school		1 (20%)
College 4 years or more		2 (40%)
Income		
Below 10,000		2 (40%)
10,000–30,000		1 (20%)
30,000–70,000		2 (40%)
Marital status		
Married		4 (80%)
Divorced		0 (0%)
Widowed		1 (20%)
Never married		0 (0%)
Household membership		
Lives alone		0 (0%)
Lives with spouse or partner		4 (80%)
Lives with family members/caregiver		1 (20%)
Living situation		
Rent apartment/condo		0 (0%)
Owns house/townhouse		5 (100%)
Houseless		0 (0%)

*N* = 12, Mean (M), Standard deviation (SD), sample size (*n*), Percentage (%).

At one-year post WY PACE closure, nearly one-half of all participants indicated having no friends to talk with (*n* = 5, 42%), and one-quarter of all participants reported having no relatives to talk with (*n* = 3, 25%), based on Berkman-Syme Social Network Index scores. In addition, a majority (*n* = 11, 92%) indicated that they had no participation in church or other clubs/organizations. Participant social support scores according to the MSPSS, were indicative of moderate social support (*M* = 4.0, *SD* = 4.2), with moderate social support demonstrated in the Significant Other domain (*M* = 4.5, *SD* = 1.95) and in the Family domain (*M* = 4.8, *SD* = 1.9). Meanwhile, low social support was indicated in the Friends domain (*M* = 2.8, *SD* = 1.7). Therefore, participants experienced adequate social support from their families and significant others but had limited support within their friendships. Loneliness scores from the R-UCLA among members of the sample ranged from 48–58, with the mean score indicative of high levels of loneliness (*M* = 54.4, *SD* = 2.9).

Similarly, health status was assessed using the SF-36 at the time of the interview. Participants’ scores on the SF-36 suggested overall poor health status. Specifically, participants’ general health (*M* = 39.2, *SD* = 21.2), physical functioning (*M* = 10.4, *SD* = 11.2), limitations due to physical health (*M* = 25.0, *SD* = 33.7), energy levels/fatigue (*M* = 22.5, *SD* = 28.5), pain level (*M* = 52.7, *SD* = 35.7), emotional well-being (*M* = 47.3, *SD* = 21.8), social functioning (*M* = 34.4, *SD* = 33.3), and limitations due to emotional problems (*M* = 36.1, *SD* = 43.7) were calculated. According to original normative data presented by Ware (30), these scores are commensurate with a categorization of health status as “poor”.

Depressive symptoms were examined at the time of the WY PACE closure, based on scores presented in the final Plan of Care, and at one year follow-up, by self-report. The mean depressive symptoms scores, one-year following the WY PACE closure, were indicative of moderate levels of depressive symptoms (*M* = 9.4, *SD* = 3.2), with 41.7% (*n* = 5) experiencing mild symptoms, 33.3% (*n* = 4) experiencing moderate symptoms, and 25% (*n* = 3) experiencing severe levels of depression. This is significantly higher than depressive symptoms scores at the time of the WY PACE closure (*M* = 5.5, *SD* = 3.3, *p* = 0.001), where 83.3% (*n* = 10) experienced normal or mild symptoms of depression, and only 16.7% (*n* = 2) experienced moderate or severe levels of depression (see Table 4).

**Table 4:** Paired samples *t*-test for the geriatric depression scale at the time of the WY PACE closure and one-year follow-up from former WY PACE participants.

Time point	*M*	*SD*	*t*	*p*
At WY PACE closure	5.50	3.32		
One-year follow-up	9.42	3.20	−3.93	0.001

*N* = 12, Mean (M), Standard deviation (SD), T-statistic (t), *p*-value (*p*).

### 3.2 Thematic analysis

Three areas of inquiry from the interview were examined: (1) the impact of the WY PACE closure (i.e., how WY PACE closure affected social isolation and loneliness); (2) how participants have adjusted to the loss of services; and (3) recommendations to address concerns of social isolation and loneliness.

#### 3.2.1 Impact of PACE closure on social isolation and loneliness

Participants universally described that the closure of WY PACE led to feelings of loneliness and social isolation. Many participants described feeling disconnected and withdrawn from their community and deprived of social support and contact with others. Many stated that WY PACE was their primary, or only, social outlet, and its closure led to the experience of a social void. Three themes emerged from the data analysis on the impact of the program’s closure on social isolation and loneliness: (1) *Depressed, Anxious, and Lonely*; (2) *No Transportation to Anywhere*; and (3) *Chopping Off a Part of Life*.

##### 3.2.1.1 Depressed, anxious, and lonely

All participants described a reduction in their mental and cognitive well-being that came about after the WY PACE closure, which they attributed to the loss of social connections from the WY PACE program. Many participants described feeling depressed, anxious, and hopeless about their situation. These feelings were experienced by individuals regardless of sex or risk for nursing home admission. For example, a typical response was mentioned by a female participant (aged 85 to 89) from a low-income household who lives alone with multiple chronic illnesses and was classified as being at no immediate risk for nursing home admission with normal cognitive status. She said, “*I have never been lonely in my life until after PACE… I do not like being lonely and miserable. I do not like it. You know, when you are depressed, everything stresses you out, and some days I feel a little better now. But I was down at the bottom when PACE closed… I get stressed out easier now. In fact, I did not used to get stressed out. And I have to say this is post-PACE” (P1)*. Importantly, her value of social engagement was reflected by her primary goal at WY PACE, which was to engage in recreational activities with friends in the program. The participant reported a moderately high level of loneliness (R-UCLA = 57), higher depressive symptoms scores (Pre GDS = 3, Normal; Post GDS = 9, Moderate), and poor health status at the time of the interview.

Another participant (P2; Female) who was over 80 years of age, experiencing multiple chronic medical conditions, living with a caregiver, and was classified as being in immediate jeopardy of nursing home admission (i.e., Category 2, needing skilled nursing care within the next six months) at the time of the WY PACE closure described the benefit of the program for social engagement: *“It got me out of the house, it gave me something to do, people to talk to. And I was just, I was real sad [when it closed]. Real sad. To get up in the morning, get dressed, to feel like you are going to work, or doing something. Then it just quit. And I was just very sad” (P2).* Her goal at WY PACE was to participate in physical therapy and increase her walking stamina. Despite social support received, she reported experiencing a moderately high degree of loneliness at the time of the interview (R-UCLA = 53). The measures indicated that she also experienced an increase in depressive symptoms in the year following WY PACE closure (Pre GDS = 6, Post GDS = 8) and she reported that her health status (i.e., physical, emotional, and social functioning) was somewhat worse than one year ago.

Similar impacts on mental health were experienced by somewhat younger older adults with less disabling physical conditions. For example, a low-income woman (aged 70 to 74), who was categorized as limited to low risk for nursing home admission, also experiencing multiple chronic conditions (e.g., hypertension, chronic pain, and history of excoriation disorder) described her experience with deteriorating mental health: *“I would say the depression is higher. I would say the anxiety is higher. I’m a picker… And now, part of that’s from – it got worse after PACE but now, it’s really picked up because I do not have anywhere to live. But, you know, we [PACE] were working on that, and we were getting some progress done there, but now what?” (P7)*. The participant reported a moderately high degree of loneliness (R-UCLA = 57), no change in depressive scores (GDS = 8) since the PACE closure, and poor health status at the time of the interview.

##### 3.2.1.2 No transportation to anywhere

The second theme identified was the impact of the WY PACE closure on transportation. Participants described that they perceived their only access to socializing in both the community and at WY PACE was through the transportation system offered to them while enrolled at WY PACE. WY PACE offered transportation to all participants, whether that was to and from the PACE building or to other resources that were offered in the community. About 75% of participants described that the transportation loss from the WY PACE closure impacted their isolation, loneliness, and feelings of disconnection and estrangement from their local community.

One participant with high spiritual needs expressed that she lost her ability to go to church and connect with her church community since WY PACE closed. For example, she described, *“Transportation became the biggest problem for me…it has been a constant struggle with transportation. Just getting to the doctor is impossible…Transportation was the biggest loss since PACE closed. I can only go to certain appointments like important medical appointments…And of course, I mentioned to you the worship service, that was such a blessing. That just filled my spiritual needs because it got to where I could not find a ride to church. And now, I do not have a ride to church. Not everybody can handle an old person with a walker” (P1)*. This participant mentioned that she has very limited contact with family and has no friends since the closure of WY PACE, therefore accessibility to transportation is limited to nonexistent.

Another participant also described her spiritual needs as not being met since the WY PACE closure. Participant 5, a woman (aged 65 to 69) with multiple sclerosis and arthritis, was living alone and classified as being at high risk for skilled nursing at the time of the WY PACE closure. She described, *“Once I got sick, I could not drive anymore. So, I had nothing. Then the PACE bus came, and I got freedom back. And now it’s gone. Because there aren’t a lot of places that have the buses. And a lot of churches do not. The only way I can go to church is because the minister picks me up” (P5).* The participant reported a moderately high degree of loneliness (R-UCLA = 55), an increase in depressive symptoms (PRE GDS = 0, Post GDS = 4), and rated her physical functioning and role limitations due to her physical health as poor.

Another participant shared similar experiences with transportation loss which also impacted her social life. Participant 10, a woman (aged 65 to 69), reported that she experienced many physical limitations (e.g., hypovolemia, type 2 diabetes, and mild cognitive impairment), lived alone with ongoing home caregiver support, and was classified as being medically stable. She stated, *“Anywhere that you had an appointment or anything, the PACE bus would take you and bring you home or bring you back to PACE or whatever. I really, really liked that because now I do not have transportation to anywhere… And with PACE, I was busy with my mind and with activities and socializing. They took care of my medical, everything. I never had to wait for anything… [I learned after PACE] how lonely it was going to be not going anywhere. Because I cannot just go anywhere” (P10).* This participant reported moderate levels of loneliness (R-UCLA = 48), an increase in depressive symptoms since the time of the WY PACE closure (Pre GDS = 3, Normal; Post GDS = 10, Moderate), and indicated that her general health was poor at the time of the interview.

##### 3.2.1.3 Chopping off a part of life

The final theme of the program closure’s impact on isolation and loneliness was a substantial loss in social support from others (e.g., WY PACE staff and participants) and social opportunities provided by WY PACE. When asked about social adaptations, many said they never adapted and were stripped away from the friendships and activities that once provided them meaning and purpose. This experience was described consistently across participants, regardless of risk for nursing home admission or demographic characteristics.

One participant, a male (aged 70 to 74) living with his caregiver, classified as medically stable with ongoing caregiver support, also experienced social barriers after the closure. His caregiver stated, *“He would have stuff to talk about when he came home and about the activities they did and the people that came in, or something that happened. That’s missing now. Needless to say, we were not pleased that it closed. And there’s nothing. Right now, there is no resource. There’s no place he can go to play games for the afternoon or go play computer games or card games, or board games with people, or go see some of the people from PACE. There’s none of that. So, I mean it’s just kind of chopped off part of his life” (Caregiver of P11)*… At the time of the interview, the participant reported experiencing a moderately high degree of loneliness (R-UCLA = 55), an increase in depressive symptoms (Pre GDS = 2, Normal; Post GDS = 6, Mild), and a declined health status.

Another participant who lived alone with very limited support from family and friends also described the experiences that impacted her social world after the WY PACE closure: *“You’re going to make me cry. But you know my biggest worry is being alone. And it’s harder. And I’m a coward. And as stuff gets harder, it’s not as much fun. And it was always fun at PACE, but you could always find different ways of doing it. If you could not get it this way, then they show you a better way or they’d tell you to use your walker and you can push yourself up. And there’s nobody to show me how to do that now. And that I miss so badly” (P5).*

Similar social losses were experienced by a low-income man (aged 70 to 74) who experienced multiple chronic illnesses and was at no immediate risk for nursing home admission at the time of the PACE closure. For example, he said, *“I just do not have as much social activities or anything… meeting different people and talking to a few friends” (P6).* The participant reported a moderately high degree of loneliness (R-UCLA = 57), no changes in depressive symptoms after the WY PACE closure, and poor physical and emotional functioning.

#### 3.2.2 Adaptations to the PACE closure

To investigate how participants have adjusted to the loss of services that address social isolation, questions were asked regarding their current social life following the WY PACE program’s closure and how they have managed changes in their social routines since then. A recurring theme that emerged in this area of inquiry was the *Struggle to Compensate*. Within this theme, participants described a significant disruption in their social lives after the WY PACE program closure. WY PACE was a safe space where older adults could depend on others in the program and the community, and once that was taken away, participants felt they could not adjust to their new circumstances. Routines, roles, and responsibilities were greatly changed after the WY PACE closure, resulting in difficulty adjusting and functionally adapting to their new “normal”.

A spousal caregiver of a Hispanic male participant, classified as medically stable with ongoing caregiver support, described what her husband’s current social world looked like following the WY PACE closure: “*What social world? That’s what I mean. Now it’s just him and I. No contact with anyone…and I cannot imagine the people who live alone and what they are going through now. It’s just so heartbreaking because they loved it and they smiled, and some would knit bags and sewed bags for their walkers and attached them to their walkers and they would make sure everyone who had a walker had a bag or they knitted something warm for them. They all had something to do, and they liked helping each other. And now they are all alone and I just cannot imagine” (Caregiver of P3).* The participant reported a moderately high degree of loneliness, moderate symptoms of depression, and a poor health status.

Another caregiver described a participant’s inability to socially compensate after the WY PACE program. This Hispanic male (aged 70 to 74) was living with his caregiver and was classified as being at immediate risk for nursing home placement within the next six months at the time of the WY PACE closure. His caregiver stated, *“He does nothing. Just zero. Zero. And I feel really guilty because I do not do enough with him. But on a typical day, it’s more, yeah, it’s just sit and watch TV. Yeah, not very stimulated” (Caregiver of P12).* The participant reported experiencing a moderately high degree of loneliness (R-UCLA = 55), an increase in depressive symptoms (Pre GDS = 6, Mild; Post GDS = 14, Severe), and a poor health status during the interview.

The inability to socially engage was also experienced by others. One participant (P9; Male) aged 75 to 79, experienced multiple chronic medical conditions, and was classified at WY PACE closure as being in immediate jeopardy of nursing home admission, had a caregiver who described his social life since the WY PACE closure. *“His social world now is very poor. He will not go outside and sit in the sunshine anymore. He will not ever. He goes outside once in a while to take out the trash. That’s it. That’s once in a while… I think he feels lonely honey, with me, you bet. He only has me” (Caregiver of P9).* Participant 9 also experienced a moderately high degree of loneliness (R-UCLA = 52) at the time of the interview, an increase in depressive symptoms (Pre GDS = 7, Mild; Post GDS = 15, Severe), and his caregiver reported that his health status (i.e., physical, emotional, and social functioning) was somewhat worse than one year ago.

At the time of the interview, another participant, living alone with multiple chronic conditions, was not deemed at immediate risk of nursing home admission but shared insights into the impact of the WY PACE program closure on her social life and that of other participants. The woman (aged 80 to 84) described, *“Well, there is not much of a social life. I do not do much, but I just try to do the best I can… But some of the participants had to go to a nursing home because there was nowhere else to go and they were very unhappy about it and so were their families, but they felt like they did not have any other choice” (P4).* The participant reported a moderately high level of loneliness (R-UCLA = 55), an increase in depressive symptoms (Pre GDS = 4, Normal; Post GDS = 12, Severe), and no changes in health status.

#### 3.2.3 Recommended actions

Participants were asked about recommendations they had to address the issues of social isolation and loneliness that they experienced after the WY PACE closure. The following three themes emerged: *Reopening WY PACE, Connection to Existing Community Resources, and Fostering Social Engagement*.

##### 3.2.3.1 Reopening PACE

All participants suggested that the WY PACE program should ultimately reopen, allowing all former participants to regain the routines that they found nourishing and fulfilling. Some participants suggested that if WY PACE could not reopen, it would be beneficial to create similar programs, or a smaller WY PACE program with fewer participants, or expand to other areas in Wyoming. For example, Participant 6 stated, *“I think they ought to just try and bring it back. They had that big building all renovated and stuff. It’s a waste… there’s a lot of other stuff that are available for the younger people that have particular problems and stuff, but not us” (P6).*

Participant 10 had similar ideas. She said, *“They just need to open it again. I mean, I know it costs a lot of money to run it, but what are they going to do? Just put us in a building and let us all die? That’ll happen sometime in the future” (P10).* She expressed concerns about the community not taking care of the older adult community and neglecting their need for care and support.

Participant 4 also suggested that the WY PACE program should be reopened. She said, *“My biggest recommendation is they reopen PACE, even if it’s—even if they have shorter hours or less providers, it would be something… Just bring PACE back please. We need it” (P4).*

Participant 7 suggested that if WY PACE could not reopen, there could be different ways to implement care programs like PACE in Wyoming. She described, *“I think that there needs to be more outreach or whatever with them [senior programs], you know? Because will PACE come back or will it not? You know? You at least have to have – you have got something that you can build on, you know? You could turn them into quasi-PACEs” (P7).*

##### 3.2.3.2 Connection to existing community resources

Many participants recommended some form of linkage to existing community resources. For example, the caregiver of Participant 12 mentioned ideas about reintroducing Participant 12 to community resources. For example, he said, *“Even just respite… just a place that you can drop off, give them some social [time]—even if you do not want to pay for the medical care but you’ll save a ton of money if you do, but even if you do not want to pay for it, then okay, at least give them a place to hang out. Other than the senior center… They need a little more care than the senior center” (Caregiver of P12).*

The caregiver of Participant 11 also expressed similar needs. For example, she stated, *“We drastically need some sort of respite program. Both for seniors, like a senior activity center that is adequately staffed, so that… well it would be open so many days a week they could go and play cards or play games or whatever under supervision. That would be good for him. For caregivers it would be nice to have some sort of respite program like that, that would take the burden off of us so that we would not have to be so responsible all the time. That is one of my biggest suggestions is to have some sort of a respite program” (Caregiver of P11).*

Participant 2 expressed the need for a transportation service for older adults to get them to appointments and social opportunities in the community. She stated, “*What I really think, all these people that do not have ways to get around they cannot drive, like me, they need transportation to pick them up and to take them home” (P2)*.

##### 3.2.3.3 Fostering social engagement

In the final theme of recommended actions, many participants expressed a need for substantial support in order to engage in social programs, whether that was someone facilitating a reunion for the WY PACE participants or assisting in social activities. The caregiver of Participant 3 mentioned the opportunity of a reunion for the former WY PACE participants, encouraging social connection with each other. For example, she said, *“That would be great if we could go and visit the participants. He made a lot of friends there and spent so many good times with them. I would not mind doing that for him” (Caregiver of P3).*

Participant 2 suggested activities for older adults that facilitated social involvement. For example, she stated, *“They could even have a movie day, and they could pick you up and take you just like that. You know, going to the rodeo out that way. They would take you out there. I think a program doing some type of senior activities, for those that cannot get out on their own, having someone come pick them up, and having someone there to help with going to the bathroom and taking them to do an activity and socializing” (P2).*

Participant 4 suggested a few social activities as well as a reunion with former WY PACE participants. She described*, “Probably some kind of group outings… Like a Picnic at the park. Like everybody could bring something if they are able to…like to have something of a reunion of some sort… Just to drive around to do something” (P4).*

## 4 Discussion

Qualitative findings from this retrospective outcomes evaluation one year after the closure of the WY PACE program showed that former participants experienced increases in isolation and loneliness and increases in feelings of depression. The loss of both the WY PACE Day Center and the previously guaranteed transportation services appeared to influence these experiences. Former participants and caregivers described that the day services at WY PACE not only provided recreational activities such as games, physical exercise, arts and crafts, and social interaction but also facilitated transportation to the facility and community events. The discontinuation of these services had a profound impact on social connection and participants’ sense of purpose. Results also showed that former WY PACE participants were unable to adapt to the loss of social relationships by forming new connections in the community. To our knowledge, this is one of the first studies to document the impact of a PACE closure in regard to social isolation and loneliness.

Our findings align with established research literature. For example, many studies have examined the relationship between loneliness, social isolation, and depression (5, 36, 37). Specifically, researchers have proposed that experiencing loneliness and isolation tends to precede the onset of depression (38). In many cases, social connection is perceived to be a protective factor against depression (39); our evaluation adds to this literature and suggests that the reduced social contact and interaction resulting from the closure of WY PACE may have exacerbated depressive symptoms among participants. Additionally, other studies have highlighted the detrimental impact of social isolation on various aspects of health and well-being. For instance, social isolation has been associated with an increased risk of heart disease, stroke, and cancer (40, 41), as well as a heightened risk of cognitive impairment and neurocognitive disorders such as Parkinson’s disease and Alzheimer’s disease (42, 43).

Several explanations are offered in the literature for why isolated and lonely older adults may also experience increases in depression. Friendships are a significant component of mental well-being, offering a different type of social connection compared to familial or spousal relationships (44). Additionally, research indicates that social opportunities offer a space for older adults to connect with others who share similar interests or life experiences (45). Therefore, the removal of these social opportunities can impose a significant burden on mental well-being. Furthermore, research suggests that transitions and losses can exacerbate social isolation and loneliness, potentially leading to increased symptoms of depression (46). The closure of WY PACE serves as an example of these transitions and losses impacting participants’ social and emotional well-being.

The qualitative results may also be interpreted within the framework of existing theory, specifically, Self-Determination Theory. According to this framework, when psychological needs are satisfied, individuals experience increased motivation to engage in activities that hold personal significance, consequently leading to a higher quality of life (47). Conversely, unmet needs may lead to psychological distress and diminished mental well-being (48). The Self-Determination Theory posits three innate psychological needs—autonomy, competence, and relatedness—that promote intrinsic motivation and determination in one’s life (49). In this evaluation, participants identified two psychological needs—autonomy and relatedness—that were fulfilled when they were at WY PACE. For example, the WY PACE program was designed to provide opportunities for older adults with serious illnesses to live autonomously in the community and socially connect with others (14). The closure of WY PACE resulted in a loss of autonomy and relatedness, as participants no longer could connect meaningfully with others, potentially contributing to the current experience of depressive symptoms. Results of this evaluation illustrated the function of Self-Determination Theory among WY PACE participants who experienced the closure of their program. For example, the PACE program was designed to provide opportunities for older adults with serious illnesses to live autonomously in the community and to optimally “age in place” for as long as possible (14). Participants also expressed responsibility for the tasks and activities they were recommended to do as part of their individualized plans of care at PACE. Although some participants depend on their caregivers for their health care needs, they expressed having more control over their schedule and goals of care in PACE than they do now. When PACE was removed, participants expressed that their autonomy in making choices regarding their medical and social needs was also taken away. Further, the psychological need for relatedness was stripped from participants. For example, participants reported that the loss of PACE meant they no longer had an outlet to connect with others and be meaningfully involved with their friends, the PACE staff, or the local community.

Qualitative findings in this evaluation were one of the first to identify several potential solutions for addressing isolation and loneliness among older adults with chronic conditions, such as those who were enrolled in WY PACE. The primary recommendation from former WY PACE participants was to reopen a PACE program in their area or have a similar program available to older adults. All participants had expressed a need for a program that ensured access to medical, behavioral, and social services. PACE is a unique model that blends medical care, day services, and social interaction opportunities for community-dwelling older adults. Due to the cost of this comprehensive approach, this exact model would be hard to replicate without dedicated PACE funding. However, there are other funding sources that could address the most salient gaps in services for older adults, particularly those which provide access to socialization and transportation services. Title III of the Older Americans Act provides funds dedicated to supporting congregate meals, transportation to medical appointments, and evidence-based health promotion programs, including those to address the effects of social isolation and loneliness. In communities experiencing the closure of PACE or similar programs, stakeholders representing federal funding agencies (i.e., State Units of Aging), local healthcare systems, community-based organizations, and program participants could collaborate to creatively identify strategies and funding sources to address the resultant gaps in services (50). According to Wang and colleagues (51), the collaboration of researchers, policymakers, and community members can effectively produce and maintain programs that serve the older adult community. The use of community advisory boards has been identified as an effective approach to engaging stakeholders in intervention development and sustainability (52). Researchers have suggested that comprehensive medical programs are beneficial for ensuring quality care for older adults with chronic illnesses (53, 54). However, programs like these and PACE have been underutilized or have lost the funding needed to maintain these services (22). Further work is needed to maintain and support care models that are similar to PACE.

Potential programs to address isolation and loneliness among older adults have also been identified in recent research literature. For example, the CARELINK Program (55) is one solution, offering weekly home visits and tailored assessments to effectively manage social isolation. This program may serve as a valuable alternative for former WY PACE participants and individuals eligible for PACE, providing support for those who face challenges in independently engaging in community-based social activities. The results of the study conducted to examine the efficacy of the CARELINK program showed promise in bridging the gap from isolation to engagement. Nurses from CARELINK were shown to motivate the older adult participants to seek health promotion activities in the community and socially integrate more with their neighbors and friends. Consequently, the authors found that social isolation decreased significantly in older adults when implementing this model. This model may be valuable for reaching and supporting people who qualify for PACE, but do not have such a resource.

In addition to home-based programs, senior centers are a common and widely available community-based resource that promote social engagement for all older adults, including those who may be lonely or have lower social support (56). Choi and McDougall (57) found that older adults who attended senior centers had lower depressive symptoms, better coping skills, and more friendships than homebound individuals. Further, to promote participation, senior centers will often provide their own transportation services or are often located in areas where public transportation is easily accessible (58).

If implementing a program similar to PACE is not possible, participants indicated a desire for some form of linkage to community resources. Community resources are vital in promoting healthy aging and fostering social engagement among older adults (59). Given that primary care providers often function as the only point of contact for many older individuals, these settings offer an opportunity to connect socially isolated older adults with community resources (60). Nevertheless, a gap exists between primary care providers and surrounding community resources, resulting in missed opportunities to effectively connect with older adults (60). Boll et al. (61) addressed the gap between primary care providers and community resources by introducing primary care liaisons (PCLs) hired by an Area Agency on Aging. These PCLs utilized diverse outreach strategies, such as presenting to clinical teams to educate providers about available community services. The study demonstrated that primary care liaison models effectively enhance the linkage between primary care providers and community resources, supporting optimal care and social engagement for older adults. Further research should explore various referral processes and methods to enhance clarity in referring socially isolated older adults.

Finally, participants indicated a need for substantial, tangible support for engagement in social programs. Other researchers have identified that older adults desire more social programs (62, 63). The same is true of this evaluation’s participants; however, they indicated a need for more support and facilitation prior to engaging in these social programs. Many of the participants in this evaluation also presented functional limitations in activities of daily living and instrumental activities of daily living; therefore, their needs may differ from those of other older adults.

Several options may exist to promote social engagement for older adults with functional limitations. For example, adult day programs are community-based services designed to support older adults with chronic illnesses through health monitoring, psychosocial support, and socialization opportunities (64). Extensive research conducted by Dabelkno-Schoeny and King (65) as well as Sadarangani et al. (66) have investigated the utilization of adult day services. Their findings consistently highlight the positive experiences reported by participants, emphasizing the establishment of meaningful social connections and assistance with managing functional limitations. Future research in this area should specifically examine the impact of adult day services on social isolation and loneliness among older adults with chronic conditions.

Participants also expressed a desire for community support, such as volunteer-driven transportation, to help them venture into the community. Initiatives like the Dakota Area Resources and Transportation for Seniors (DARTS) in Minnesota provide tailored transportation solutions for older adults who are isolated and lonely (67). DARTS provides a range of transportation options, including individual rides, contracted group rides, and weekly semi-fixed loops. These services utilize buses and vans designed to accommodate older adults who may experience functional limitations. Inclusive transportation programs like DARTS may be a solution that provides support to older adults with functional limitations to socially engage with their community. Future research should examine programs like DARTS in terms of facilitating social engagement for older adults with functional limitations.

While there are multiple options that have the potential to maintain social engagement, few, if any, of these options have been tested. Future research should prioritize evaluating interventions similar to those listed above at the time of any new PACE closure to assess their effectiveness in supporting the social and health needs of older adults. In addition, prior to the closure of future programs, decision-makers may engage in a closure planning process to ensure all components of PACE, including socialization, is completed to mitigate potential consequences for participants such as social isolation and loneliness. This process could involve an environmental scan of existing social programs that may assist in such a transition, ensuring that participants have access to necessary social support services during and after the closure (68).

### 4.1 Limitations

This evaluation was among the first to examine how the WY PACE closure impacted social isolation and loneliness in former participants, to understand social adaptations following the WY PACE closure, and to identify needs and preferences for future interventions addressing social isolation and loneliness. However, there are potential limitations to be addressed. First, the sociodemographic composition of the sample (i.e., being predominantly White, female, and cisgender), while consistent with Wyoming’s population (69), may not generalize to program closures of individuals with more diverse memberships. In addition, not all former WY PACE participants were able to engage in the evaluation. The primary reason for nonparticipation in this evaluation was loss of follow-up (i.e., inability to contact participants). Despite reaching out to all potential participants, a considerable number had disconnected telephone numbers. Other participants were living with cognitive impairment and were unable to participate. Furthermore, the emotional nature of the subject may have introduced potential biases in participants’ retrospective reflection and subsequent responses. For instance, participants might have overemphasized the negative impacts due to the emotional distress caused by the program closure. Likewise, while two time points were available to measure depressive symptoms, this study did not have retrospective ratings on social isolation and loneliness prior to the WY PACE closure, thereby making it difficult to assess changes in loneliness and isolation following the program closure. This absence of pre-post data may have also introduced bias and highlights the need for future studies to collect comprehensive baseline data before program closures occur. Despite this limitation, qualitative data suggest that participants perceived the closure of WY PACE as having an influence on changes in social isolation, loneliness, depressive symptoms and health status. Should future PACE programs close, attempts should be made to gather quantitative data prior to a program closure and at follow-up to help determine whether a change in social isolation, loneliness, and health status has occurred from the time of closure. Despite these limitations, the research presented in this evaluation has characterized the experience of former WY PACE participants in response to the loss of program support. The evaluation also identified perceived needs, concerns, and recommendations to address the problem of social isolation and loneliness among older adults affected by the WY PACE closure. These findings highlight the need for intervention strategies that tackle social isolation and loneliness in older adults with chronic illnesses.

## 5 Conclusion

In summary, this evaluation has provided valuable insight into the personal experiences of former WY PACE participants and the impact participants believed that the closure had on their social lives. Further research is needed to understand what occurs when important support programs close and the impact of the closures on participant social isolation and loneliness. Moving forward, public health policies should prioritize the development and maintenance of comprehensive programs like PACE that address the complex needs of older adults with chronic illness, including needs for social engagement. As our findings suggest, alternative programs like CARELINK and other community-based resources may play a vital role in addressing social isolation and offer valuable insights for policymakers aiming to enhance the well-being of older adults with chronic conditions in the absence of programs such as WY PACE. New programs may also be developed to buffer the impacts of program loss on older adults’ social lives. This evaluation emphasized the need for proactive strategies that foster social engagement and combat the detrimental effects of program closures on this specific demographic.

## Data Availability

The raw de-identified data supporting the conclusions of this article will be made available by the authors, without undue reservation.
